# Preparation and Characterization of Efficient and Safe Rotenone Solid Nanodispersion by Self-Emulsifying Technique

**DOI:** 10.3390/nano15141056

**Published:** 2025-07-08

**Authors:** Yunfei Zhang, Xuesheng Lin, Yunlong Qian, Mingda Qin, Shujing Zhang, Lanying Wang, Yanping Luo

**Affiliations:** School of Tropical Agriculture and Forestry, Hainan University, Haikou 570228, China; zhangyunfei2020@hainanu.edu.cn (Y.Z.); 20095132210106@hainanu.edu.cn (X.L.); 22220951320076@hainanu.edu.cn (Y.Q.); 21210904000018@hainanu.edu.cn (M.Q.); 990992@hainanu.edu.cn (L.W.)

**Keywords:** solid nanodispersion, self-emulsifying, rotenone, photostability, bioactivity

## Abstract

Self-emulsifying solid nanodispersion technology is emerging as an attractive strategy to prepare new eco-friendly and efficient nano-formulations due to its simple, energy efficient and easy scale-up process. However, it is still unknown whether this technology can be employed to cope with the drawbacks of botanical insecticides including poor water solubility, rapid photodegradation and limited targeting efficiency. In this study, rotenone (Rot) was selected as a model of botanical insecticides, and its solid nanodispersion (Rot–SND) was prepared by a self-emulsifying method combined with parameter optimization. Our target nano-formulation, consisting of 5% Rot, 20% surfactant complexes of 8% Ethylan 992 and 12% EL–80, and 75% lactose, exhibited excellent storage stability and significantly improved the pseudo-solubility of Rot by at least 250 times. The average particle size and polydispersity index (PDI) of Rot–SND were determined to be 101.19 nm and 0.21, respectively. Rot–SND displayed smaller contact angles and greater retention on both cucumber and cabbage leaves than those of a commercial emulsifiable concentrates (ECs). Rot–SND was also more resistant to photodegradation, with a degradation rate reduced by 27.01% as compared with the ECs. In addition, the toxicity of Rot–SND towards *Aphis gossypii* was 3.01 times that of the ECs, with a median lethal concentration (LC_50_) of 1.45 µg a.i./mL. Under the field conditions, Rot–SND showed a prolonged duration for *A. gossypii* control, with a significantly higher control efficacy (88.10%) on the 10th day than that of the ECs (77.02%). Moreover, a 2.34-fold decline in the toxicity towards nontarget mosquito larvae was observed for Rot–SND as compared with the EC. Overall, for the first time, our results indicate the role of Rot–SND as an eco-friendly and efficient way to improve the solubility, foliar affinity, photostability, bioactivity and eco-safety of Rot. This research also provided a feasible strategy to prepare more eco-friendly botanical pesticide formulations of high efficiency.

## 1. Introduction

Botanical bioinsecticides are emerging as a non-toxic promising alternative to cope with the growing concerns of synthetic pesticides regarding their adverse effects on the environment and human health [1,2]. Despite extensive studies successfully demonstrating the high potency of various botanicals for pest insect control, botanical insecticides in current commercial use, however, are still limited due to a lack of physicochemical stability, fast photodegradation and the resultant poor field performance [2,3]. Thus, it has become a key topic to focus on the development and application of known botanicals by innovative formulation strategies, rather than to discover more pesticidal plants and identify new insecticidal substances [1,2,3]. As a well-known isoflavone botanical insecticide, rotenone (Rot) exhibits good insecticidal activity against a broad spectrum of pest insects in 137 families, including aphids [4], and it is recognized as a green pesticide safeguarding crop production in organic farming systems [4,5,6]. Yet, Rot is poorly water soluble and easily degraded by exposure to light in the environment with a short duration, which severely limits its utilization efficiency by using traditional formulations in the fields [5,7]. Also, traditional Rot formulations, like emulsifiable concentrates (ECs) and microemulsions, generally contain non-eco-friendly organic solvents or excessive surfactants, which reduces the product safety as green pesticides. Although Rot is considered as a safe bio-pesticide for domestic animals and humans since it does not accumulate in the environment due to rapid degradation [8], many studies also reported its high toxicity to domestic animals, fish, and mice by direct exposure [8,9], and its role as a potential risk factor for the onset of neurodegenerative conditions [10]. Therefore, developing new botanical pesticide formulations that are of high efficiency and low ecological toxicity is a major challenge for agricultural production [2,3].

A combination of nanotechnological approaches and botanical insecticides have paved a way to develop more new formulations with high efficiency and low environmental risks, and are the leading edge of commercial development for existing botanical insecticides [3,11,12]. Currently, microencapsulation technologies based on natural polysaccharides [4,13] and nano-packaging technologies of carrier adsorption [5], crosslinking self-assembly [7] and co-precipitation [14] are reported to improve the formulation performance of Rot, including reducing its toxicity to nontarget organisms and enhancing its efficacy against target pests. However, there is only a very limited number of studies focusing on the development of Rot nano-formulations as eco-friendly pesticides of high efficiency. Chen et al. (2016) found that ZnO and SiO_2_ nanoparticles could improve Rot photostability effectively by simple mixing of them and Rot [15]. Mesoporous silica nanoparticles were used to encapsulate Rot to improve its stability and insecticidal activity [5]. Chitosan derivatives of oleoyl-carboxymethyl chitosan [16], deoxycholic acid carboxymethyl chitosan [17] and *N*, *N*-dimethylhexadecyl carboxymethyl chitosan [18] have been employed to enhance Rot solubility by forming nano-micelles. Chitosan–graphene oxide nanocomposites were also described to increase Rot solubility [6]. Further, Rot was encapsulated in biodegradable chitosan-based nanoparticles to produce a nanopesticide with enhanced bioavailability and photostability [7]. Bidyarani and Kumar (2019) prepared a Rot-loaded zein nano-formulation showing excellent antimicrobial activity [19]. Nevertheless, more work needs to be performed to develop highly efficient and eco-friendly Rot formulations using new nanotechnological approaches.

Recently, solid nanodispersion based on self-emulsifying technology (SESND) has become a new nanotechnology that requires a simple and energy-efficient formulation process with no organic solvent residues. SESND has been widely used to prepare nano-formulations of various pesticides such as emamectin [20], emamectin benzoate [21,22,23,24], avermectin B_2_ [25], lambda-cyhalothrin [26], pyraclostrobin [27] and the binary complexes of prochloraz and azoxystrobin [28]. These prepared solid nanodispersion (SND) formulations can significantly improve the water solubility, photostability, foliar affinity and bioactivity of active ingredients. However, limited studies described the capacity of SESND for the preparation of botanical insecticide formulations. It is also not known whether SESND can be used to produce a Rot nano-formulation or whether the formulation can improve the solubility, foliar affinity, photostability and bioactivity of Rot.

In the present study, a Rot solid nanodispersion (Rot–SND) was prepared by using the self-emulsifying method, and its compositions were optimized by an orthogonal experiment method. Then, the characteristics of the target Rot–SND were determined and compared with a traditional EC formulation in terms of morphology, particle size, contact angle and retention on leaf surfaces, photostability, aphicidal activity under indoor and field conditions, and toxicity towards nontarget organisms. This is the first example known to the authors that has proved the technical feasibility of SESND for the preparation of a new Rot–SND of high efficiency, which can also be extended to developing eco-friendly and efficient nano-formulations for other botanical insecticides.

## 2. Materials and Methods

### 2.1. Materials

Rotenone (Rot) standard (98%) and Rot technical material (95%) were purchased from Shanghai Aladdin Biochemical Technology Co., Ltd. (Shanghai, China). Surfactant Ethylan992 was from Nanjing Jierun Sci. Tech. Co., Ltd. (Nanjing, China). Surfactant EL–80 was from Jiangsu Haian Petrochemical Plant (Haian, China). Lactose (98%) and galactose (98%) were obtained from Macklin Chemical Technology Co., Ltd. (Shanghai, China). Ethyl acetate (99%) and sodium benzoate (99%) were obtained from Sun Chemical Technology Co., Ltd. (Shanghai, China). Commercial Rot emulsifiable concentrates (ECs, 2.5%) were obtained from Guang Nong Yao Ye Co., Ltd. (Guangzhou, China). Chromatographic-grade acetonitrile was purchased from J&K Scientific Ltd. (Beijing, China).

### 2.2. Preparation of Rotenone Solid Nanodispersion (Rot–SND)

Rot–SND was prepared by a self-emulsifying method followed by carrier adsorption and solidification [20,27]. For a standard procedure, 50 mg of Rot was dissolved in 5.0 mL ethyl acetate to prepare solution A with vortex oscillation. Then, 0.20 g of the selected surfactant was accurately weighed and added to solution A to obtain solution B with vortex oscillation. Finally, solution B was added to the lactose carrier (0.75 g), fully mixed, and dried in an oven at 45 °C for 3 h to obtain the 5% Rot–SND.

### 2.3. Orthogonal Design of Experiments

A series of one-factor-at-a-time pre-experiments were conducted to prepare a formulation of 5% Rot–SND. A binary surfactant mixture of Ethylan 992 and EL–80 was screened out as suitable surfactant complexes, and the quality of Rot–SND was determined to mainly depend on the three key factors of the weight ratio of Rot to surfactant (Rot/surfactant), the mixing proportion of Ethylan 992 and EL–80 (Ethylan 992/EL–80), and the carrier type. Thus, based on the results of our pre-experiments, orthogonal experimental design was adopted to optimize the formulation compositions of 5% Rot–SND using a L_9_(3^4^) orthogonal table designed by SPSS 21.0 software (SPSS Inc., New York, NY, USA). Each factor had three different levels (Table 1), and a blank column would be used as the fourth factor to calculate the error according the orthogonal design theory [29,30]. A total of 9 formulations were designed and tested, with the compositions listed in Table S1. The average particle size and PDI (polydispersity index) were selected as the quality indicators of the prepared formulations to perform the range analysis to determine the significance levels of the influencing factors and obtain the optimal level of each factor. The K value, as well as the range value (R) was calculated for range analysis. All the tests were repeated for three times and then averaged.

### 2.4. Particle Size and Size Distribution Measurements

The particle size and size distribution of 5% Rot–SND formulations were determined by photon correlation spectroscopy using a Malvern dynamic light scattering (DLS) instrument of Zetasizer Nano ZS90 (Malvern Instruments Ltd., Worcestershire, UK) at a fixed angle of 90°. Each Rot–SND sample was diluted into deionized water containing 0.2‰ (*w*/*w*) active ingredient of Rot and measured at 25 °C. The results were expressed as the *Z*-average (diameter) obtained from three measurements (10 runs each) with a corresponding standard deviation. The particle size and its distribution were presented as cumulant mean diameter and PDI, respectively.

### 2.5. Zeta-Potential Measurements

The Zeta potentials of 5% Rot–SND formulations were determined by measuring the direction and velocity of droplet movement in the applied electric field using a Malvern Zetasizer Nano ZS90 (Worcestershire, UK) equipped with a 4 mW He–Ne Laser (633 nm) and folded capillary cells with gold electrodes. The were recorded and analyzed by the Dispersion Technology Software 5.1 in General Purpose mode and with automatic measurements settings using the Smoluchowsky mathematical model. The samples were diluted with deionized water at the ratio of 1:250 (*w*/*v*), and each sample solution was injected directly into the chamber of a Nano-ZS90 particle electrophoresis instrument for Zeta potential analysis at 25 °C.

### 2.6. Storage Stability Evaluation of Rot–SND

The storage stability of Rot–SND was evaluated according to a previous protocol method [20]. Briefly, Rot–SND samples were placed into brown glass bottles (10 mL). After sealing, the sample bottles were placed in a fridge at 4 °C for 7 days, or in an oven at 54 °C for 14 days. The entire storage process was carried out under natural light. The particle size and PDI of Rot–SND before and after storage were determined by the DLS method as mentioned above.

### 2.7. Crystalline State Analysis of Rot–SND

The crystalline state of Rot–SND was characterized by a Brucker D8 Advance powder X-ray diffractometer (XRD, Karlsruhe, Germany) equipped with a Cu Kα source (0.154 nm, 40 kV, 40 mA). Data were recorded in an angular range of 10–80° (2*θ*) with a scan rate of 0.02°and a time step of 0.1 s.

### 2.8. Morphology Observation

For morphology observation, Rot–SND suspension was prepared and dropped onto a piece of monocrystalline silicon (0.5 cm × 0.5 cm). After being air-dried overnight, the silicon slices were then gold-coated using a Hitachi E1010 sputter coating machine (Tokyo, Japan) for 60 s, and imaged using a Hitachi S4800 cold field scanning electron microscope (SEM, Tokyo, Japan). To determine the size of Rot–SND, the SEM images were analyzed using ImageJ (https://imagej.nih.gov/ij/ (accessed on 7 November 2024)) and the diameters of at least 200 randomly selected nanoparticles were measured.

### 2.9. Contact Angle and Retention Measurements

Hydrophilic cucumber (*Cucumis sativus* L.) leaves, as well as hydrophobic cabbage (*Brassica oleracea* L.) leaves, were selected for evaluating the dynamic contact angles of Rot–SND samples. Briefly, fresh cucumber and cabbage leaves from 3-week plants were carefully cleaned to remove foliar surface dust and then fixed on glass slides. Then, 5 µL of each SND sample solution containing 0.2‰ (*w*/*w*) Rot was dropped onto a prepared leave slide and the contact angle was recorded on a JC2000D2M contact angle instrument (Zhongchen Digital Technic Apparatus Company, Ltd., Shanghai, China) using a five-point fitting method. The contact angles of commercial Rot ECs (2.5%) on fresh cucumber and cabbage leaves were also recorded as controls. Each sample was repeated for five times.

Retention on cucumber and cabbage leaves was tested by a previously described method [20,30] according to weight difference before and after impregnation. First, Rot–SND and ECs were diluted with ultra-pure water to prepare aqueous dispersions with 0.2‰ (*w*/*w*) active ingredient. Second, the initial weight of each leaf sample was recorded as M_0_ (mg) and its surface area (S_m_, cm^2^) was also measured. Then, each leaf sample was fully immersed into the prepared dispersion for 10 s, and its weight after immersion (M_1_, mg) was recorded when no droplets were sliding off the surface. Five repeated measurements were arranged for each sample. Retention (R_m_, mg/cm^2^) was calculated by Equation (1) as followed:

R_m_ = (M_1_−M_0_)/S_m_, (1)

### 2.10. Determination of Rot–SND Photostability

The photostability of Rot–SND was measured according to a previously described method [7,31], using technical Rot and its ECs as the controls. Rot–SND and ECs were dispersed in pure water to prepare formulation sample solutions containing 500 mg/L of Rot. Technical Rot was dissolved in chromatographic acetonitrile to prepare a Rot acetonitrile solution at a concentration of 500 mg/L. Quartz tubes with 1 mL sample solution were exposed to a 365 nm ultraviolet (UV) lamp (30 W, Philips) at a straight distance of 20 cm. Samples were taken off from the UV light after 24 h radiation. The Rot residue in each sample solution was determined by high-performance liquid chromatography (HPLC) analysis. All experiments were carried out for three times.

The Rot content was measured by an Agilent 1260 HPLC system (Agilent Technologies, Santa Clara, CA, USA) equipped with a C_18_ column (5 μm, 4.6 mm × 150 mm) at 25 °C. A mixture of acetonitrile and water (60:40, *v*/*v*) was used as the mobile phase with a flow rate of 1 mL/min. The injection volume was 10 µL, and the UV detector wavelength was set to 299 nm.

### 2.11. Bioassays

Laboratory aphicidal activity bioassay was carried out to test the toxicity of Rot–SND against *Aphis gossypii* using a previously described leaf-dipping method [32]. Colonies of *A. gossypii* were collected from cucumber plants and raised in our laboratory at 25 ± 1 °C, 70 ± 10% relative humidity (RH), and in a photoperiod of 16 h light and 8 h dark. A series of sample solutions of Rot–SND were prepared by dissolving it into distilled water at different Rot concentrations of 0.5, 1.0, 2, 4, and 8 µg/mL. Fresh cucumber leave disks of 6 cm diameter were dipped in a sample solution for 15 s and then air dried naturally. Leave disks with Rot–SND were further placed upside down in 90 mm Petri dishes (one disk per Petri dish), with each bottom lined with a sheet of 9 cm moist filter paper. Apterous *A. gossypii* adults of identical size and age were selected and transferred onto the treated leaves (ten individuals per leaf). Each Petri dish was then covered with a layer of transparent plastic wrap with pinholes, and incubated in a growth cabinet at identical conditions. Distilled water and Rot ECs were used as blank control and commercial formulation control, respectively. Aphid mortality was checked after 48 h incubation, and the median lethal concentration (LC_50_ value) was calculated using the probit package of SPSS 23.0 software (SPSS Inc., Chicago, IL, USA). Three leaves were arranged for each treatment group, and every assay was repeated three times.

Field aphicidal efficacy evaluation was carried out in a wolfberry tree plantation of Qingtongxia City, Ningxia Province, China (longitude: 106.17, latitude: 38.15). The Chinese wolfberry trees were grown in experimental fields under routine management. According to the field trial protocol for insecticides against aphids [33], experimental plots were randomly arranged with three repetitions and ten trees per treatment. To avoid edge effect, 2 m wide protection lines were arranged between plots. The Rot concentration of the sprayed Rot–SND dispersion was set to 200 µg/mL and the sprayed volume was 37.5 mL/m^2^, which is consistent with the field recommended dose of the commercial Rot EC (75 g/ha) by conventional volume spray (375 L/ha). Commercial Rot ECs (2.5%) and water were used as the commercial insecticide control and blank control, respectively. The control efficacy was calculated by Equations (2) and (3):

PRR (%) = (N_0_ − N_t_)/N_0_ × 100, (2)

where PRR represents the population reduction rate, N_0_ represents the initial aphid count, and N_t_ represents the aphid count after treatment.

Control efficacy (%) = (PRR_t_ − PRR_0_)/(100 − PRR_0_) × 100, (3)

where PRR_t_ and PRR_0_ represent the population reduction rates in a treated plot and in a blank plot, respectively.

The toxicity of Rot–SND towards nontarget *Aedes aegypti* larvae was calculated according to the standard procedure recommended by the World Health Organization (WHO) [34]. A laboratory strain of *A. aegypti* (Hainan strain) was provided by the Laboratory of Pesticide Science of Hainan University (Haikou, China) and used for all experiments. Rot–SND was dispersed in dechlorinated water to prepare stocks with Rot concentrations of 10, 40, 80, 100 and 150 mg/mL, respectively. Thirty-fourth instar larvae were placed into 90.0 mL of dechlorinated water, followed by adding 10 mL of a stock solution. Commercial Rot ECs and water were used as the commercial insecticide control and blank control, respectively. The larvae mortality was recorded after 48 h exposure under identical conditions and calibrated by Abbott’s method when necessary [35]. Each experiment was replicated five times. The virulence curves and LC_50_ values, as well as their 95 confidence intervals, were calculated according to the probit package of SPSS 23.0 software (SPSS Inc., Chicago, IL, USA).

### 2.12. qRT-PCR Analysis of Mitochondrial Gene Expression

Quantitative real-time PCR (qRT-PCR) assay was used to detect variations in the expression of 11 mitochondrial protein-coding genes (MtPCGs) of *A. gossypii* after Rot–SND treatment. Aphids were treated with Rot–SND at a Rot concentration of 1.45 µg/mL (LC_50_ value) for 48 h, as described in Section 2.11. Live aphid samples (100 individuals) were collected and used for total RNA extraction using TRIzol reagent (Invitrogen, Carlsbad, CA), according to the manufacturer’s instructions. The concentration and purity of RNA samples were assessed by a Thermo Scientific Nanodrop Lite Spectrophotometer (Wilmington, NC, USA) using an A260/A280 ratio ranging from 1.8 to 2.0 and an A260/A230 ratio > 2.0 as the standards of qualification. The cDNA was synthesized from the total RNA samples using an All-in-One First-Strand cDNA Synthesis Kit (Servicebio Technology Co., Ltd., Wuhan, China) and stored at –20 °C until use. MtPCGs primers were designed using a Primer Premier 5 software (Palo Alto, CA, USA) according to the complete mitochondrial genome sequences of *A. gossypii* (GenBank accession number: KJ669654) [36], and the optimal primers are present in Table S2. qRT-PCR was then performed on a Thermo Scientific QuantStudio 5 Real-time PCR System (Foster, CA, USA) using SYBR Green as the fluorescent intercalating dye. The PCR programs are present in Table S3. For normalization, the 18S rRNA gene (*18S*) was used as the endogenous control [37]. Distilled water treatment was used as the blank control, and a commercial Rot EC treatment was used as the positive control. Each gene was analyzed three times and its relative expression levels were calculated using the 2^−ΔΔCt^ method.

### 2.13. Statistical Analysis

SPSS 23.0 software (SPSS Inc., Chicago, IL, USA) was used to test for statistical significance (*p* < 0.05) by one-way ANOVA analysis and Student’s test.

## 3. Results and Discussion

### 3.1. Optimization of Rot–SND Recipe Parameters

The results of our pre-experiments revealed that combined surfactants of Ethylan 992 and EL–80 were beneficial to preparing Rot–SND with excellent dispersion. Ethylan 992 is an alkoxylated short fatty alcohol belonging to alcohol ethoxylates, a class of eco-friendly surfactants of excellent biodegradability [38,39,40]. Likewise, EL–80 is a castor oil polyoxyethylene ether that belongs to a readily biodegradable substance with very low environmental risks [41,42]. These two surfactants may adsorb on the hydrophobic solid Rot surface by their hydrophobic alkane chain to form a larger steric hindrance using the hydration of their hydrophilic polyoxyethylene chains [26]. Then the thickening of the hydration adsorption layer can reduce both the Hamaker constant and the Van der Waals attraction potential energy between particles [26,43], which can enhance the spatial repulsive force to prevent particle aggregation. The steric hindrance between particles is closely related to the hydrophilic–lipophilic balance of surfactants, which is easily obtained by regulating the ratio of combined surfactants [26,44]. From the results of our pre-experiments, we also found that three carriers of lactose, galactose, and sodium benzoate were also feasible to prepare Rot–SND due to their eco-friendliness, low cost, and potential hydrogen bond interactions with Rot, leading to their wide use in SND preparation [20,24,26,27]. Therefore, based on the discussion above, a L_9_(3^4^) orthogonal experimental design was conducted to optimize the main composition parameters of Rot–SND in terms of Rot/surfactant (factor A), Ethylan 992/EL–80 (factor B), and carrier type (factor C). Particle size and polydispersity index (PDI) are two key factors of pesticide nano-formulations [20,24,30] and are used as the evaluation indicators. Visual analysis of the effects of different factors on particle size and PDI is illustrated in Table 2. According to the R-value of particle size, carrier type was determined to be the most significant factor, followed by Rot/surfactant and then Ethylan 992/EL–80, and there was no significant interaction between the factors due to the minimum R-value of the error term. The variance analysis of particle size further indicates that carrier type and Rot/surfactant significantly affect the particle size of Rot–SND (Table S4). The R-value of PDI; however, reveals that the effect of the three test variables on the size distribution, in decreasing order, is as follows: Rot/surfactant > carrier type > Ethylan 992/EL–80. Furthermore, significant interaction exists between the test factors, with a median R-value of the error term (Table 2). As the Rot/surfactant decreased from 0.33 to 0.20, the Rot–SND PDI reduced by 25.8% and 32.3%, respectively. The factor of Rot/surfactant was also demonstrated to significantly affect the PDI of Rot–SND by variance analysis (Table S5). Based on the K value of particle size (Table 2), the Rot–SND formulation of the minimum particle size is A3B2C1 (122.80 nm). Moreover, this formulation also exhibited a minimum PDI value of 0.19 and is regarded as the optimal formulation by the K value analysis of PDI.

**Figure 1:**
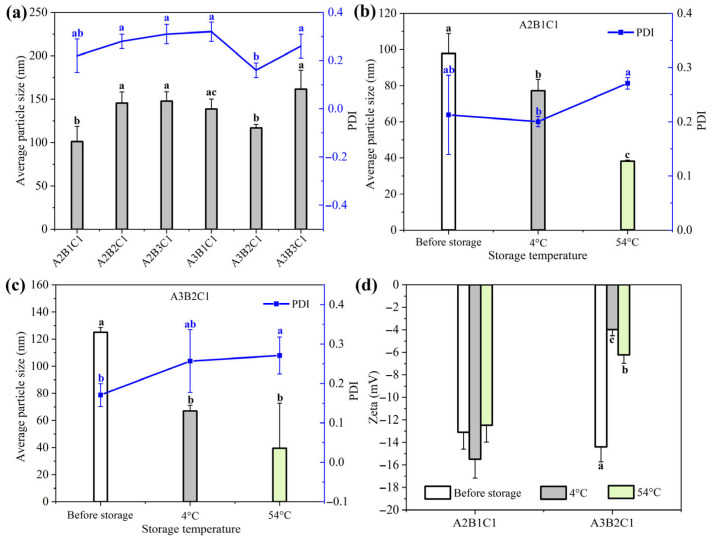
Characteristics of different Rot–SND formulations under different conditions. The average particle size and polydispersity index (PDI) of (**a**) six Rot–SND formulations at 25 °C before storage, (**b**) A2B1C1 before and after storage at different temperatures, and (**c**) A3B2C1 before and after storage at different temperatures. The Zeta potentials of (**d**) two Rot–SND formulations of A2B1C1 and A3B2C1 before and after storage at different temperatures. The blue lines in plots represent PDI vslues. The error bars represent averages and standard deviations. Different letters in the plot demonstrate significant differences at *p* < 0.05.

Based on the results of the orthogonal experiments, lactose is the suitable carrier and increasing the mass ratio of Rot to surfactant benefitted the dispersion of Rot–SND. Thus, we next measured the particle size and PDI of six Rot–SND formulations of different Rot/surfactant (1:4 and 1:5) and Ethylan 992/EL–80 (4:6, 5:5 and 6:4) ratios using lactose as the carrier, with the results illustrated in Figure 1a. Two formulations of A2B1C1 and A3B2C1 exhibited comparable particle size and PDI values, and their particle size values were significantly lower than those of the other four formulations. Storage stability is also an important characteristic of a pesticide formulation [20]. Therefore, we further determined the storage stability of A2B1C1 and A3B2C1 using particle size, PDI and Zeta potential as indicators. As shown in Figure 1b, when stored at 4 °C for 7 days, the particle size of A2B1C1 significantly decreased to 77.17 nm, with no significant change in the PDI value. Likewise, the particle size of A3B2C1 was also significantly reduced (67.01 nm), with no distinct difference in the PDI value after storage at 4 °C for 7 days (Figure 1c). After 14-day storage at 54 °C, a reduced particle size of 38.49 nm and non-significant change in the PDI value were observed for A2B1C1 (Figure 1b), while A3B2C1 exhibited not only a reduced particle size (39.56 nm) but also an increased PDI value (0.27) (Figure 1c). The reduced particle size after storage may be attributed to particle reorganization induced by Rot recrystallization in solid Rot–SND. Previous studies indicate that Rot was encapsulated in biodegradable polymer microcapsules in an amorphous state by a solvent evaporation method [13,45]. Since the biodegradable polymers are rich in hydroxyl and carboxyl groups, we speculate that Rot–SND might also contain a small part of large particles of amorphous Rot due to uneven solvent evaporation and hydrogen bonding interactions among Rot, surfactants and the lactose carrier. In crystalline carriers, including lactose, the drug may also precipitate in an amorphous form with high dissolution [46], which may also provide evidence for the existence of amorphous Rot particles in Rot–SND. The small part of large amorphous Rot particles in Rot–SND can dominate the light scattering signal and mask the presence of smaller particles, resulting in a higher particle size during DLS analysis [24,47,48]. Amorphous solid dispersion (ASD) exists in a higher energy state, which makes it more susceptible to recrystallization, a transition to a lower energy state, during storage [46,47,49]. This amorphous-to-crystalline transition can be accelerated at high temperatures due to the high molecular mobility [46,48,50]. Thus, large amorphous Rot particles increased the initial particle size of Rot–SND. Afterwards, the large amorphous Rot particles transformed spontaneously into smaller crystalline Rot particles stabilized by surfactants and the lactose carrier without overgrowth in size, during storage. High-temperature storage promoted the mobility of rotenone molecules in Rot–SND, which can accelerate the recrystallization process, further generating smaller particles. Moreover, high-temperature storage may also promote the phase transition of Ethylan 992 and EL–80 as polyoxyethylene surfactants [51], which can also accelerate the recrystallization process by phase separation [46,49]. The smaller particle size of A2B1C1 stored at 54 °C than that at 4 °C (Figure 1a) also indicates the accelerating effects of high temperature on Rot recrystallization in solid Rot–SND. A similar trend was also reported for the storage of avermectin B2 SND [25]. Notably, there were no significant changes observed in the Zeta potential of A2B1C1 after 7-day storage at 4 °C and 14-day storage at 54 °C, whereas the Zeta potentials of A3B2C1 were significantly reduced after storage (Figure 1d). Compared with A3B2C1, A2B1C1 exhibited a higher storage stability with non-significant changes in PDI and Zeta potential after storage at different temperatures. The crystal states of A2B1C1 before and after storage at 4 °C for 7 days and at 54 °C for 14 days were also demonstrated to remain unchanged, as observed by the XRD powder diffractograms (Figure S1), again indicating its excellent stability.

Taken together, A2B1C1 was selected as the optimal formulation, with a small particle size (101.19 nm), narrow size distribution (PDI = 0.21), and excellent stability, and it was used for subsequent experiments. Finally, a target Rot–SND was produced with the optimal formulation and its compositions were determined as follows: 5% Rot, 8% Ethylan 992, 12% EL–80 and 75% lactose.

### 3.2. Morphology and Particle Size of the Target Rot–SND

**Figure 2:**
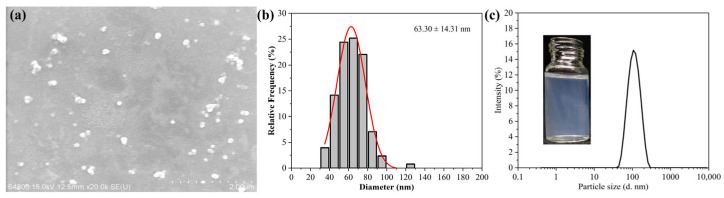
The morphology and particle size distribution of the target Rot–SND formulation (A2B1C1). (**a**) SEM image, scale bar: 2 µm, (**b**) size distribution and Gaussian fitting curve (red line) counted from SEM images, and (**c**) size distribution measured by DLS. Insert: the apparent state of Rot–SND dispersion at a Rot concentration of 500 mg/L.

The morphology of the target Rot–SND was observed by a scanning electron microscope (SEM), with the results shown in Figure 2a. The SEM image shows that the Rot nanoparticles are approximately spherical in shape with good dispersion. The average particle size based on 200 particles from the SEM images was calculated as 63.30 nm, with particle diameters ranging from 36.54 to 126.15 nm (Figure 2b). The mean particle size measured by DLS was 101.19 nm, with a narrow and single peak size distribution ranging from 43.8 to 255 nm (Figure 2c), which is larger than that obtained from the SEM images. This discrepancy can be attributed to the fact that the DLS measurement reflects the hydrodynamic diameter of the particles with a lactose hydration layer as the swollen corona, but the SEM images present the size of the dispersive particles in the dry state, without the hydration corona [20,24,52]. Moreover, the light scattering signal may be dominated by larger particles or aggregates in the dispersion, which may mask the presence of smaller particles and can result in a higher measured value during DLS analysis [24,53,54]. Therefore, in Rot–SND, the carrier lactose can not only improve the particle dispersion in water but can also limit the growth of particles in solution and during the drying process. The insert of Figure 2c shows that Rot–SND dispersion has a clear transparent and light-blue pseudo-solution appearance at a Rot concentration of 500 mg/L, which is 250-fold higher compared with Rot solubility in water (2 mg/L) [18,55,56]. This result indicates that Rot–SND can significantly increase Rot water solubility with no organic solvents.

### 3.3. Wettability and Retention of Rot–SND

**Figure 3:**
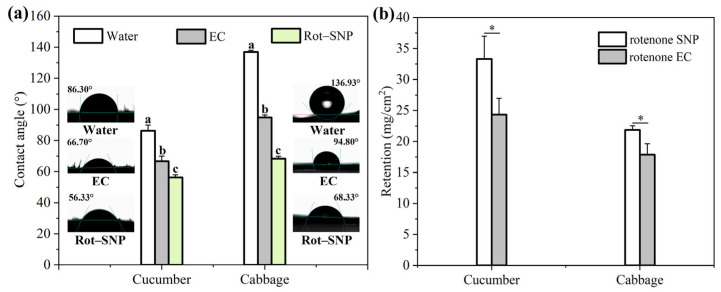
Wettability and retention of different Rot formulations. The contact angle (**a**) and retention (**b**) of Rot–SND, ECs, and water on cucumber (*Cucumis sativus* L.) and cabbage (*Brassica oleracea* L.) leaves. The error bars represent averages and standard deviations. Different letters and asterisks (*) in plot represent significant differences at *p* < 0.05.

The wetting and spreading behavior of pesticide formulations can be intuitively observed by the contact angle of pesticide droplets on the target surface, which is related to their surface tension and interface properties [20,26]. Consequently, the contact angle of the Rot–SND dispersion droplets was measured on cucumber (*Cucumis sativus* L.) and cabbage (*Brassica oleracea* L.) leaves, respectively. As shown in Figure 3a, the contact angle of ultra-pure water was measured to be 86.30° on hydrophilic cucumber leaves, and 136.93° on hydrophobic cabbage leaves. The contact angles of the commercial Rot ECs on cucumber and cabbage leaves were determined to be 66.70° and 94.80°, respectively. The respective contact angle of Rot–SND on cucumber and cabbage leaves was measured to be 56.33° and 68.33°, smaller than those of the Rot EC formulation (Figure 3a). As supported by the literature [20,26], formulations with a small particle size can enhance the contact area with leaves due to their large special surface area.

The retention of Rot–SND on cucumber and cabbage leaves was also explored, with the results shown in Figure 3b. The retention of Rot–SND on cucumber leaves was determined to be 33.30 mg/cm^2^, which was 1.37 times that of the commercial Rot ECs (24.33 mg/cm^2^). Also, the retention of Rot–SND on cabbage leaves was measured to be 21.85 mg/cm^2^, which was 1.22 times that of the commercial ECs (17.88 mg/cm^2^). These results indicate that Rot–SND with a small particle size has excellent wettability, showing a smaller contact angle and greater retention on hydrophilic and hydrophobic leaves as compared with a commercial EC.

### 3.4. Photostability of Rot–SND

**Figure 4:**
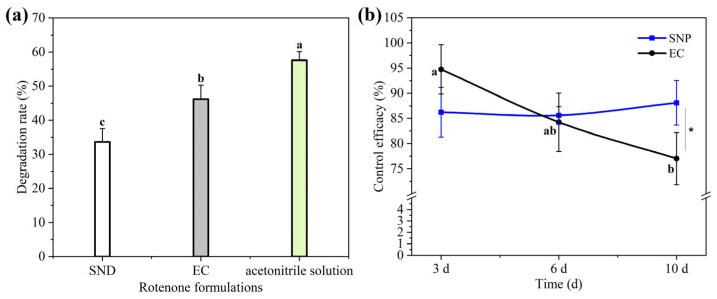
(**a**) Photodegradation rate of three Rot formulations under UV radiation. (**b**) Field efficacy of Rot–SND and ECs against *Aphis gossypii* at different time intervals after spaying. The error bars represent averages and standard deviations. Different letters and asterisks (*) in plot represent significant differences at *p* < 0.05.

Rot readily degrades by exposure to sunlight in fields and loses bioactivity, so it is of great importance to preserve its bioactivity by reducing photodegradation [5,7]. Photoprotectants [31] and nanoencapsulation [5,7] have been reported to enhance the photostability of Rot. For instance, the photodegradation rate of abamectin in SND was proved to be much lower than that of a commercial EW (emulsion in water) formulation [20,21]. Thus, we tested the photodegradation rate of Rot in Rot–SND under UV radiation. As shown in Figure 4a, 33.70% of Rot degraded in Rot–SND, whereas 46.20% and 57.60% of Rot degraded in ECs and acetonitrile solution, respectively, after UV radiation for 24 h. The photodegradation rate of Rot in Rot–SND was much lower than that in EC. The preparation process of the SND involved drying liquid or semi-solid self-emulsifying ingredients onto soluble powders, allowing active ingredients to disperse in a solid hydrophilic matrix as particles or in microcrystalline form. As the crystalline phase [57,58,59] and particle state [20,21] have been reported to be more stable than the amorphous phase under photo-exposure, Rot–SND improved the photostability of Rot probably due to its existing state in lactose matrix.

### 3.5. Indoor Toxicity of Rot–SND Against Aphis gossypii

Previous studies have revealed that SNP could improve the solubility, foliar affinity and photostability of pesticides, and finally result in higher bioactivity [20,21,24,26,27]. From the discussion above, Rot–SND has many advantages over commercial EC. It still needs to be confirmed whether these advantages substantially make a difference in the bioactivity of Rot–SND. Based on this, the toxicity of Rot–SND to *A. gossypii* was evaluated in the laboratory and compared with a commercial EC. As shown in Table 3, both Rot formulations are highly toxic to *A. gossypii*, with the median lethal concentrations (LC_50_) lower than 4.50 μg a.i./mL. Compared with EC, Rot–SND exhibited a higher aphicidal activity against *A. gossypii,* with an LC_50_ value of 1.45 µg a.i./mL, significantly smaller than that of the ECs (4.36 µg a.i./mL). From the results of our studies on foliar wettability and retention, we deduced that the excellent foliar wettability and retention of Rot–SND could have increased its foliar adhesion and penetration, which play a key role in enhancing the effective action dose of Rot when contacting and feeding aphids, and in turn increased their mortality [21].

### 3.6. Field Efficacy of Rot–SND Against A. gossypii

Though the high bioactivity of SND has been vastly verified under laboratory conditions [20,21,22,23,24,25,26,27,28], its performance in the field is scarcely discussed. Thus, to further explore the potential of Rot–SND in practical applications for aphid control, we tested its aphicidal activity against *A. gossypii* in a wolfberry tree plantation. As shown in Figure 4b, these two Rot formulations exhibited high aphicidal activities with a minimum control efficacy of 77.02% in the 10 days after spraying. Of note, the control efficacy of Rot–SND demonstrated an increasing trend over time in spite of a lack of statistical significance between the time intervals, whereas an obvious decreasing trend was observed for the control efficacy of the ECs that decreased from 94.75% to 77.02% within the period from the 3rd day to the 10th day. The control efficacy of Rot–SND was slightly lower than that of the ECs without significant difference on the 3rd day, comparable to that of the ECs on the 7th day, but significantly higher than that of the ECs on the 10th day (Figure 4b). Within the first 3 days, the permeation of organic solvent in the ECs and lower Rot photodegradation can explain its slightly higher control efficacy at this stage. With continuous exposure in the fields, the control efficacy of the ECs began to decline continuously due to the solvent evaporation and the enhancement of photodegradation. For Rot–SND, water evaporation might benefit its efficacy exertion, probably due to the decreased particle size inducing strong adhesion and penetration. Compared with the hydration particle size of Rot–SND observed by DLS (Figure 2c), the dry Rot–SND of smaller particle size was determined by SEM (Figure 3b), indicating particle size reduction during water evaporation. The result of the indoor bioassay on cucumber leaves coated by the dry Rot–SND exhibited a higher aphicidal activity (Table 3) and also provided evidence for the key role of a small particle size on bioactivity. The reason for the prolonged and stable control efficacy of Rot–SND is multi-faceted and involves its nanoparticle size, excellent foliar wettability and retention, and resistance to photodegradation. A similar pattern was also reported for a nano-formulation of pyrethrin [52]. All in all, these results demonstrate that Rot–SND can both improve Rot utilization efficiency and prolong its duration, which provides evidence for its potential in aphid control practices.

### 3.7. Effect of Rot–SND on the Mitochondrial Gene Expression in A. gossypii

**Figure 5:**
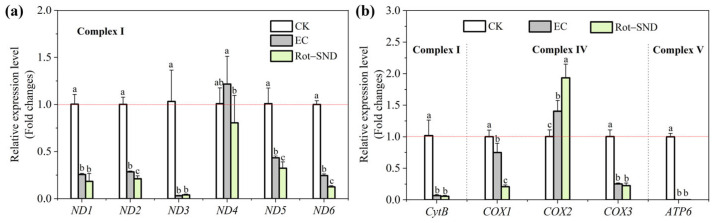
Impact of Rot–SND on the expression levels of 11 mitochondrial protein-coding genes (MtPCGs) in complex-I (**a**) and complexes III, IV and V (**b**) of *A. gossypii* after 48 h exposure at a rotenone concentration of 1.45 μg/mL. qRT-PCR was used to detect the fold changes in MtPCG expression levels compared to each internal control (*18S*). Distilled water and a commercial Rot ECs were used as the blank and positive controls, respectively. The error bars represent averages and standard deviations. Different letters on the plot represent significant differences at *p* < 0.05.

Rot is reported to cause a strong inhibition of mitochondrial complex-I to block the respiratory electron transport chain in insects, and to result in mitochondrial dysfunction and a reduction in ATP supply [10,60]. The *A. gossypii* mitogenome is known to encode 13 subunits of respiratory complexes I, III, IV, and V, including seven NADH dehydrogenase subunit genes (*ND1*–*ND6*, *ND4l*), one Cytochrome b gene (*CytB*), three Cytochrome c oxidase subunit genes (*COX1*–*COX3*) and two ATP synthase subunit genes (*ATP6* and *ATP8*) [36,61]. Therefore, we assessed the effect of Rot–SND on the expression of 11 mitochondrial protein-coding genes (*ND4l* and *ATP8* were excluded due to their short sequence length) in *A. gossypii* using qRT-PCR, to verify the high efficiency of Rot–SND as an insecticidal formulation targeting mitochondria. The results show that nine mitochondrial protein-coding genes (MtPCGs) were significantly down-regulated by both Rot–SND and the commercial ECs at the same rotenone concentration of 1.45 μg/mL, whereas *COX2* was significantly up-regulated by these two rotenone formulations (Figure 5). *ND4*, however, was not significantly altered by either Rot–SND or the ECs (Figure 5a). Although Rot–SND and ECs exhibited similar regulatory patterns for MtPCGs, the effect of Rot–SND was more significant than that of EC, especially on *ND2*, *ND4*, *ND5*, *ND6*, *COX1* and *COX2* (Figure 5). These findings not only reveal the effect of the Rot formulations on *A. gossypii* mitochondrial gene expression but also provide evidence for the higher aphicidal activity of Rot–SND relative to the commercial EC.

### 3.8. Toxicity of Rot–SND Toward Nontarget Mosquito Larvae

Pesticides can cause severe ecological and environmental problems after entering aquatic environments by drift, leaching, and run-off during field applications [62]. Rot has been proven to be highly toxic to aquatic animals [63], including fish [4] and mosquito larvae [64]. *Aedes aegypti* larvae serve as a model to conduct toxicity assessments of environmental pollutants [65,66]. Thus, we further tested the toxicity of Rot–SND toward nontarget mosquito larvae. As shown in Table 4, the toxicity of Rot–SND to *A. aegypti* larvae was determined to be significantly lower than that of Rot EC, with an LC_50_ value of 8.79 μg a.i./mL, which is 3.34 times that of Rot ECs(2.63 μg a.i./mL). The direct contact effect of ECs and their organic solvent components may enhance the toxicity of this formulation, while the surface lactose coating effect of Rot–SND and the hydration layer may act as attenuated factors. Overall, Rot–SND shows improves ecological safety by both a lower exposure risk to nontarget aquatic organisms and a reduced application dosage due to its increased bioactivity against target organisms.

## 4. Conclusions

In this work, Rot–SND was prepared and optimized to obtained an optimal formulation consisting of 5% Rot, 20% complex surfactants of Ethylan 992 (8%) and EL–80 (12%), and 75% lactose as a carrier by a self-emulsifying technique. Rot–SND demonstrated a series of improved properties including uniform and small particle size, high storage stability, superior foliar wettability and retention, low photodegradation rate, enhanced bioactivity towards target aphids, prolonged efficacy in the field, and reduced toxicity towards nontarget mosquito larvae, as compared with commercial Rot ECs. The water solubility of Rot in Rot–SND increased by 250 fold relative to the technical Rot. The photodegradation rate of Rot–SND was reduced by 27.01% relative to the ECs. Rot–SND exhibited a higher aphicidal activity, with an LC_50_ value reduced by 2.00 times compared to that of the EC. Rot–SND also showed a prolonged duration in the field, with a significantly higher control efficacy on the 10th day than that of the ECs. Further, the toxicity of Rot–SND towards nontarget mosquito larvae was reduced 2.34 times compared to that of the ECs. Consequently, the prepared Rot–SND could be an efficient formulation to significantly promote the utilization efficiency of Rot with improved ecological safety. The strategy used in this study can also be extended to other botanical pesticides to prepare more eco-friendly formulations of high efficiency.

## Figures and Tables

**Table 1:** Variables and their levels for preparation of 5% Rot–SND.

Levels	Variables		
	Rot/Surfactant ^1^ (A)	Ethylan 992/EL–80 (B)	Carriers (C)
1	1:3	4:6	Lactose
2	1:4	5:5	Galactose
3	1:5	6:4	Sodium benzoate

^1^ Surfactant represent a binary mixture of Ethylan 992 and EL–80.

**Table 2:** Visual analysis of particle size and PDI results of Rot–SND.

Serial Number	Factors ^1^				Particle Size (nm)	PDI
	A	B	C	D		
1	1	1	1	1	161.70 ± 3.81	0.25 ± 0.06
2	1	2	2	2	181.80 ± 2.46	0.29 ± 0.02
3	1	3	3	3	293.60 ± 6.85	0.40 ± 0.02
4	2	1	2	3	141.20 ± 8.43	0.24 ± 0.04
5	2	2	3	1	233.10 ± 14.13	0.22 ± 0.04
6	2	3	1	2	152.10 ± 9.69	0.23 ± 0.04
7	3	1	3	2	239.60 ± 10.23	0.22 ± 0.05
8	3	2	1	3	122.80 ± 6.94	0.19 ± 0.03
9	3	3	2	1	138.70 ± 6.78	0.20 ± 0.02
Particle size						
k1	212.37	180.83	145.53	177.83		
k2	175.47	179.23	153.90	191.17		
k3	167.03	194.80	255.43	185.87		
R	45.33	15.57	109.90	13.33		
PDI						
k1	0.31	0.24	0.22	0.23		
k2	0.23	0.24	0.24	0.25		
k3	0.21	0.28	0.28	0.28		
R	45.33	0.04	0.06	0.05		

^1^ A: Rot/surfactant; B: Ethylan 992/EL–80; C: carrier type. Surfactant represent a binary mixture of Ethylan 992 and EL–80.

**Table 3:** Toxicities of Rot–SND and ECs against *Aphis gossypii* ^1^.

Formulations	Regression Curve	LC_50_ (μg a.i./mL)	95% Confidence Limit	χ^2^
Rot–SND	y = 0.67x − 1.16	1.45 ± 0.48	0.88–2.23	0.48
EC	y = 0.72x − 0.46	4.36 ± 0.87 *	2.88–6.42	0.73

^1^ LC_50_ represents the median lethal concentration after 48 h exposure and is presented as the mean ± standard deviation of three independent repeated experiments. The active ingredient (a.i.) represents rotenone. χ^2^ represents the chi-square value of a curve equation. The toxicity regression curve, 95% confidence limit and χ^2^ are presented as the integrated results of three independent repeated experiments. Asterisk (*) represents significant difference at *p* < 0.05.

**Table 4:** Toxicities of Rot–SND and ECs against early fourth-instar *Aedes aegypti* larvae ^1^.

Formulations	Regression Curve	LC_50_ (μg a.i./mL)	95% Confidence Limit	χ^2^
Rot–SND	y = 2.67x − 2.52	8.79 ± 0.49 *	7.42–12.22	4.01
ECs	y = 2.01x − 0.85	2.63 ± 0.44	1.41–3.95	6.19

^1^ LC_50_ represents the median lethal concentration after 48 h exposure and is presented as mean ± standard deviation of three independent repeated experiments. The active ingredient (a.i.) represents rotenone. χ^2^ represents the chi-square value of a curve equation. The toxicity regression curve, 95% confidence limit and χ^2^ are presented as the integrated results of three independent repeated experiments. Asterisk (*) represents significant difference at *p* < 0.05.

## Data Availability

The datasets generated and analyzed during the current study are available from the corresponding authors upon reasonable request.

## Supplementary Materials

The following supporting information can be downloaded at https://www.mdpi.com/article/10.3390/nano15141056/s1, Figure S1: X-ray diffraction patterns of Rot–SND before and after storage; Table S1: The orthogonal experimental design table; Table S2: Primers for qRT-PCR assay; Table S3: Amplification program for qRT-PCR assay; Table S4: Variance analysis of particle size; Table S5: Variance analysis of PDI.

## Abbreviations

The following abbreviations are used in this manuscript:

Rot	Rotenone
SND	Solid nanodispersion
Rot–SND	Rotenone solid nanodispersion
EC	Emulsifiable concentrates
