# Supplementary material for: Preparation and Characterization of Efficient and Safe Rotenone Solid Nanodispersion by Self-Emulsifying Technique
Source: Nanomaterials (Basel). 2025 Jul 8;15(14):1056. doi: 10.3390/nano15141056 (PMC12300314; doi:10.3390/nano15141056)
Supplement: Supplementary file 1 [file nanomaterials-15-01056-s001.zip › nanomaterials-3664209-supplementary.pdf]

nanomaterials

MDPI

Supplementary Materials

Preparation and characterization of efficient and safe rotenone solid nanodispersion by self-emulsifying technique

Yunfei Zhang [1], Xuesheng Lin [1], Yunlong Qian [1], Mingda Qin [1], Shujing Zhang [1,*], Lanying Wang [1] and Yanping Luo [1,*]

[1] School of Tropical Agriculture and Forestry, Hainan University, Haikou 570228, China
* Correspondence: Correspondence: sjzhang@hainanu.edu.cn (S.Z.); yanpluo2012@hainanu.edu.cn (Y.L.)

Table of contents

1. **The orthogonal experimental design table (Tab. S1)**

2. **Primers for qRT-PCR assay (Tab. S2)**

3. **Amplification program for qRT-PCR assay (Tab. S3)**

2. **Variance analysis of particle size (Tab. S4)**

3. **Variance analysis of PDI (Tab. S5)**

4. **X-ray diffraction patterns of Rot–SND before and after storage (Fig. S1)**

Tab. S1 The orthogonal experimental design table

Experiment number	Variables			
	A[a]	B[b]	C[c]	Blank column
1	50 mg/150 mg	60 mg/90 mg	Lactose 0.80 g	1
2	50 mg/150 mg	75 mg/75 mg	Galactose 0.80 g	2
3	50 mg/150 mg	90 mg/60 mg	Sodium benzoate 0.80 g	3
4	50 mg/200 mg	80 mg/120 mg	Galactose 0.75 g	3
5	50 mg/200 mg	100 mg/100 mg	Sodium benzoate 75 g	1
6	50 mg/200 mg	120 mg/80 mg	Lactose 0.75 g	2
7	50 mg/250 mg	100 mg/150 mg	Sodium benzoate 0.70 g	2
8	50 mg/250 mg	125 mg/125 mg	Lactose 0.70 g	3
9	50 mg/250 mg	150 mg/100 mg	Galactose 0.70 g	1

[a]A: Mass ratio of rotenone to surfactant; [b]B: The mixing proportion of Ethylan 992 and EL–80; [c]C: The carrier type. 50 mg of rotenone was used for preparation of 5% rotenone nanodispersion.

Tab. S2 Primers for qRT-PCR assay

Primers	Sequence (5’-3’)
ND1-F	AAGGTGAGTCAGAATTAGTTTCTGG
ND1-R	TTAATTTATCATAACGAAATCGAGG
ND2-F	TTAAATGGAATGGCAGGGTTACTA
ND2-R	TGCCAACCATATTCCTGTAGAATT
ND3-F	CCTTTTGAATGTGGATTTGAC
ND3-R	ATGATCCAAATTTTCATTCATAA
ND4-F	TGGTTGAGGTTATCAGTATGAGCG
ND4-R	CACAAATAGGAGCTTCAACATGAG
ND5-F	ATAATAGCTTCTTTAACTAAAAGAGCTC
ND5-R	TGAACTAATGAAGACACAGGAGTAGG
ND6-F	TATATTTCAAGAATTGCTTTCA
ND6-R	TTTGACGAATTGGTCCTTTA
CytB-F	TTGTAGGATATGTATTACCATGAGG
CytB-R	TAAATAAGGAATTGCTGAGAGTAAG
COX1-F	TTGATTCTTCCTGGATTTGGACT
COX1-R	TGAAGTAGGCTCGTGTATCTACATCT
COX2-F	TGCAATACCTTCCCTTCACTTACT
COX2-R	TAAGATCAAAATCATTGATGTCCAA
COX3-F	AGCCCATGACCAATCTTAATAGC
COX3-R	CCTTGAAAAGTTCTTTCTCGAATT
ATP6-F	TTAATTCCACTAAATACACCTAT
ATP6-R	AATTGATAAAGATATTGGTCG
18S-F	ATTGACGGAAGGGCACC
18S-R	CGCTCCACCAACTAAGAACG

Tab. S3 Amplification program for qRT-PCR assay

System	Procedure
2 x Q3 SYBR qPCR Master Mix (Universal) 10 µL	Predegeneration at 95ºC for 5 min
PCR Forward Primer (10 µM) 0.4 µL	Cycle nitiation:
PCR Reverse Primer (10 µM) 0.4 µL	Degeneration at 95ºC for 15 s
cDNA solution 1.0 µL	Renaturation 60ºC for 60 s
ddH_2O up to 20 µL	40 cycles

Tab. S4 Variance analysis of particle size

Source	Type III sum of square	DF	Mean square	F-value	P-value
Corrected model	79111.25	6	13185.21	24.78	0.00
Intercept	924075.00	1	924075.00	1736.78	0.00
A[a]	10454.79	2	5227.40	9.83	0.001
B[b]	1315.82	2	657.91	1.24	0.31
C[c]	67340.63	2	33670.32	63.28	0.00
Error	10641.24	20	532.06		
Total	1013827.49	27			
Corrected total	89752.49	26			

[a]A: Mass ratio of rotenone to surfactant; [b]B: The mixing proportion of Ethylan 992 and EL–80; [c]C: The carrier type. 50 mg of rotenone was used for preparation of 5% rotenone nanodispersion.

Tab. S5 Variance analysis of PDI

Source	Type III sum of square	DF	Mean square	F-value	P-value
Corrected model	0.08	6	0.01	3.41	0.02
Intercept	1.69	1	1.69	431.51	0.00
A[a]	0.06	2	0.03	7.16	0.005
B[b]	0.01	2	0.004	1.10	0.35
C[c]	0.02	2	0.008	1.97	0.17
Error	0.09	20	0.004		
Total	1.9	27			

Corrected total	0.16	26

[a]A: Mass ratio of rotenone to surfactant; [b]B: The mixing proportion of Ethylan 992 and EL–80; [c]C: The carrier type. 50 mg of rotenone was used for preparation of 5% rotenone nanodispersion.

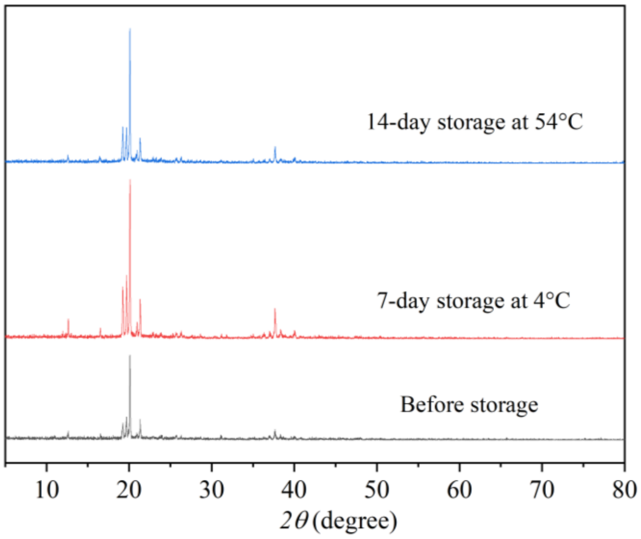

Fig. S1 X-ray diffraction patterns of Rot–SND before and after storage
